# Comparison of Input-Data Matrix Representations Used for Continual Learning with Orthogonal Weight Modification on Edge Devices

**DOI:** 10.3390/s26020425

**Published:** 2026-01-09

**Authors:** Ronald Mendez, Andreas Maier, Johannes Emmert

**Affiliations:** 1 Fraunhofer IIS, Fraunhofer Institute for Integrated Circuits IIS, Division Development Center X-Ray Technology, Flugplatzstr. 75, 90768 Fürth, Germany; johannes.emmert@iis.fraunhofer.de; 2 Pattern Recognition Lab, Friedich-Alexander Universität Erlangen-Nürnberg, Schlossplatz 4, 91058 Erlangen, Germany; andreas.maier@iis.fraunhofer.de

**Keywords:** Internet of Things, Mobile Edge Computing, distributed learning, continual learning, artificial neural twin, orthogonal weight modification

## Abstract

**Highlights:**

**What are the main findings?**

- The Fisher matrix is suitable as a complexity reduction approach, applicable for continual learning with Orthogonal Weight Modification, on edge devices.
- NEig-OWM is suitable for smaller models, typically used in distributed-process optimization approaches, such as the Artificial Neural Twin.

**What are the implications of the main findings?**

- It is possible to deploy an IIoT network of autonomously and continually learning devices.
- The distributed IIoT network can be automatically optimized though backpropagation.

**Abstract:**

The number of industrial processes in which smart devices have been employed rises every day; these devices can be found performing tasks related to the automation, digitization, or optimization of the process. Generally, for these tasks, the devices need to communicate with each other and with a central unit monitored by humans, which is where Industrial Internet of Things (IIoT) comes into play, allowing a network to be built between the devices. Communication might be enough for monitoring purposes, but the optimization and automation of the process are yet to be addressed. In this study, we use an object detection sensor as an initial test subject to explore the Artificial Neural Twin (ANT) as a distributed-process optimization tool in combination with Orthogonal Weight Modification (OWM), a continual learning (CL) method used to augment self-operating devices (i.e., microcontrollers used for machine-vision sensors) with the capacity to learn new tasks autonomously. Some of these devices lack the hardware capacity to run a CL algorithm, which also motivated the comparison of the Fisher matrix, NEig-OWM, and LoRA as matrix approximations to reduce the complexity of the operations between them. Among the compared matrices, we found the Fisher matrix to be the least expensive solution with a negligible reduction in the model’s performance after CL, which makes it a viable solution for large AI models, while NEig-OWM is better suited for smaller models that require fewer hardware resources but more control over the CL algorithm.

## 1. Introduction

The integration of smart sensors and intelligent machines into industrial processes is now a widely adopted practice. The Industrial Internet of Things (IIoT) focuses on connecting these devices for optimization and process automation purposes [1]. To that end, smart devices need to be capable of collecting, processing, and transmitting information from the environment to the network, where it is further processed and then translated into feedback by a server device that transmits back to the edge devices of the network [2].

The Artificial Neural Twin (ANT) enhances IIoT devices by enabling decentralized model predictive control setups. It integrates the principles of differentiable data fusion (for distributed data collection) and backpropagation (for distributed feedback) to support optimization and process digitization [3]. Effective processing of information gathered from both the environment and the network requires dedicated computational units within IIoT devices, typically Artificial Intelligence (AI) models, such as object detection systems, trained to execute specific tasks.

Training such models is generally time- and data-intensive, making it a major challenge for dynamic processes where tasks are constantly evolving [4]. Moreover, training a model for a new task leads to the model no longer being able to perform the former task, a phenomenon known as catastrophic forgetting [5]. In the field of continual learning (CL), many techniques have been developed for models to learn new tasks while avoiding forgetting; moreover, training data requirements can be minimized if these techniques are used in combination with the ANT as their source of training stimuli, which is a future step in our line of research. However, within the ANT network, access to the loss function is restricted, and therefore, gradient-based CL methods must be selected.

Orthogonal Weight Modification (OWM) is one such method. It prevents models from forgetting by building a matrix,

(1) $$P=I-A{\left(A^{T}A+\alpha I\right)}^{-1}A^{T},$$

that projects training gradients in the orthogonal direction to the space formed by the gradients used to learn former tasks (original tasks’ knowledge space), where I refers to an identity matrix, α is a relatively small variable, and A is the matrix formed by the gradient vectors used to learn former tasks; this means that even though new gradients can be collected within the process of learning a new task, this matrix remains constant, to be recalculated only after the mentioned task has been learned, and the preparation to learn the next task begins [6].

The problem of applying OWM in IIoT AI models lies in storing matrix A formed by the gradients produced while learning previous tasks and processing it to build the projector P. $A\in R^{nxm}$, where in a per-layer implementation of OWM, *n* refers to the length of a corresponding model layer and *m* to the number of training samples; intuitively we see that for large layers, this implementation becomes infeasible, especially within the capabilities of an edge device.

As mentioned previously, distributed systems consist of several such devices communicating with each other as part of a process. In this study we simulated a small example process, emulating the complexity of an industrial sorting facility, focusing mainly on a vision AI sensor powered by an object detection model (VGG11), which detects several types of objects on a conveyor belt and calculates the corresponding mass flow. Afterwards, we mimic the insertion of a previously unseen object, which constitutes the new task to be learned through CL.

The considered sensor represents a Mobile Edge Computing (MEC) device, which typically offers lower latency and reduced energy consumption compared to centralized IIoT architectures, but is subject to significant computational and memory constraints [7]. Considering these limitations, our objective is to investigate the impact of three alternative representations of matrix A—the Fisher Information matrix, Low-Rank Approximation (LoRA), and Null Space Eigenface (NEig-OWM)—on CL performance and computational complexity in order to identify suitable approaches for implementing OWM on resource-constrained edge devices.

## 2. Related Work

Reducing the inference and training times of machine learning models is a topic of significant interest. Model compression techniques such as quantization aim to lower the precision of calculations to minimize model inference time [8]. Pruning approaches, on the other hand, identify sections of the model (i.e., layers, neurons, parameters) whose contribution is of minimal importance for the overall performance and remove them from the model’s structure [9]. In the context of continual learning, it may appear that pruning techniques inevitably impair a model’s capacity to learn new tasks, given the removal of model sections where new knowledge can be stored.

Nevertheless, pruning remains a popular technique for improving a model’s training efficiency on edge devices. As mentioned previously, pruning minimizes the model’s available space for CL; therefore, approaches such as Task-Aware Dynamic Masking (TDM) have developed “expand-and-shrink” techniques, which initially identify the importance of model weights for the current task; a metric is then used to mask the unimportant sections of the model, which are then pruned, resulting in a sparse model. During the initial epochs of CL, new knowledge is stored in the recently freed sections of the model, and then the importance metric is calculated again, followed by the corresponding pruning, which improves the efficiency of the remaining epochs of CL given the sparsity of the model [10].

On the training-data side, TDM calculates the importance of each sample, storing only the most informative for previous and new tasks; this data contains samples from old tasks, which mitigates catastrophic forgetting, and samples from the new task, which favors learning. Along similar lines, the Latent Reply approach [11] stores information from both previous and new tasks; however, instead of retaining samples in the input space, it stores the activations of the model’s internal layers. In federated CL approaches, instead, replay data from the server is divided among the client edge devices as customized datasets [12], aiming to reduce CL latency by training with less data and then improving the model’s performance through the aggregation characteristics of federated learning.

Contrary to pruning, structural continual learning techniques aim to incorporate new sections into the model where new knowledge can be stored. The Architectural and Regularization approach (AR1) [13] extends the prediction head of the model to incorporate the new task’s labels; then, during CL, old and new model parameters are trained, ruled by a penalty term in the loss function, as applied in Elastic Weight Consolidation (EWC) [14].

The newly introduced model parameters in such approaches have been the subject of parameter reduction strategies, such as Low-Rank Approximations (LoRAs) applied to their matrix representations. This technique improves training efficiency and allows previously learned model parameters to be frozen, while training only the low-rank approximated layers [15]. Further steps towards the optimization of model training involve decomposing the weight update matrix $\Delta W\in R^{nxm}$ into two low-rank matrices ${\mathrm{LoRA}}_{A}\in R^{nxk}$ and ${\mathrm{LoRA}}_{B}\in R^{kxm}$; then, the update function is $W=W_{0}+\alpha \Delta W=W_{0}+\alpha {\mathrm{LoRA}}_{A}{\mathrm{LoRA}}_{B}$, with which the number of updated parameters during training is drastically reduced [16]. The approaches investigated in this study are used to reach a similar goal, which is to assess several matrix representations to minimize the resources required for model adaptation through CL.

## 3. Materials and Methods

Throughout this section we describe the algorithms and tools used to build the experimental setup required for the proper assessment of the tested approaches. We discuss case-specific metrics, and at last, we describe the pipeline for the test cases.

### 3.1. Artificial Neural Twin (ANT)

The ANT augments the functionality of the IIoT network by providing a platform for distributed-process optimization. Its core idea is to represent processes as neural networks (NNs), where machines correspond to the neurons and their parameters to the network weights.

**Figure 1:**
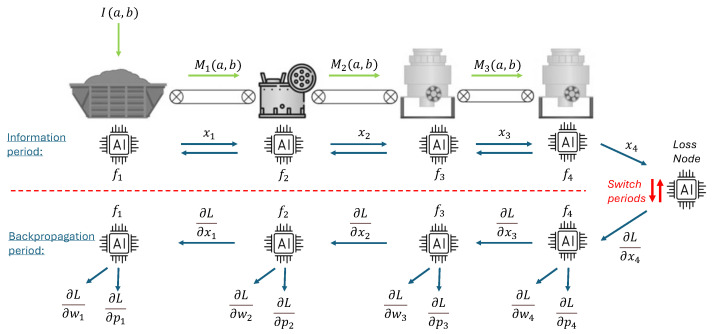
ANT forward and backpropagation periods, where $f_{i}$ refers to the model of ANT node *i*, with *i*∈{1,2,…,n}; I(a,b) the input material flow; $M_{i}\left(a,b\right)$ the output; and $M_{n}$ the process output. $\frac{\partial L}{\partial x_{i}}$ refers to the gradient with respect to the ANT node’s state $x_{i}$, $\frac{\partial L}{\partial p_{i}}$ the gradient with respect to the ANT node’s parameters $p_{i}$, and $\frac{\partial L}{\partial w_{i}}$ the gradient with respect to the model’s parameters $w_{i}$. Note: From “Towards continual learning with the artificial neural twin applied to recycling processes” by Mendez et al., 2025 [17]. Licensed under CC BY 4.0.

In the ANT, like in an NN, during the forward pass, all machines communicate their state through the network, as shown in Figure 1. Then, they reach a consensus between the network-received and internal information in the process stage (ANT node) through data fusion to update their state $x_{i}$ and to forward propagate it through the network. This allows the state to be assessed at any process stage, which is important for quality control assessments and the evaluation of the global loss function at the exit. The generated loss gradients trigger the backpropagation period, which occurs on the basis of the process and time, propagating gradients to each machine involved in the process and, within them, from future time steps to the present, which allows the optimization of the current machine’s parameters.

The ANT can receive process quality control measurements from the user [3]. This means that at any stage, production recordings can be performed and introduced into the digital process. Afterwards, this data is contrasted with the state estimate for the respective ANT node, and then gradients are calculated and accordingly backpropagated to be used as training stimuli for CL. Another source of these stimuli is generated during data fusion, where, as mentioned above, every ANT node reaches a consensus for its state, a process in which data fusion residuals are produced, which can be used by AI models to learn new tasks. These sources of training stimuli make the ANT a great ally for implementing CL in industrial process chains.

### 3.2. Stability–Plasticity Trade-Off

The main properties of a CL technique are the model’s stability and plasticity while learning new tasks. Stability reflects the model’s capacity to retain previous knowledge, while plasticity refers to the capacity to acquire new knowledge. A highly stable approach will generate a model that retains knowledge well but that hardly learns, and vice versa for a highly plastic approach. This relation is known as the stability–plasticity trade-off [18].

Stability can be evaluated in terms of the forgetting measure (FM) [19], which relates the model’s past and current performance,

(2) $$f_{j,k}=\underset{i\in 1,\ldots ,k-1}{max}\left(a_{i,j}-a_{k,j}\right),\forall j<k,$$

where $a_{i,j}$ refers to the model’s maximum performance (for this study we used F1 score as a metric) on task *j* calculated after learning task *i*, and likewise, $a_{i,k}$ is calculated after learning the new task *k*. Then, the forgetting at the *k*-th task is calculated as

(3) $$FM_{k}=\frac{1}{k-1}\sum_{j=1}^{k-1}f_{j,k},$$

from where the stability is simply calculated as

(4) $$S_{k}=1-FM_{k}.$$

The plasticity, on the other hand, refers to the model’s capacity to learn new tasks. This is evaluated in terms of its convergence on the new task,

(5) $$P_{k}=\frac{a_{k,t}}{E_{k,t}},$$

where $a_{k,t}$ corresponds to the performance right after convergence, and $E_{k,t}$ refers to the epochs necessary to reach this condition. In general, convergence to an equilibrium refers to the training state where the error has been minimized, and further epochs present minimal changes in the model’s performance, which indicates that a set of optimal parameters has been found [20]. Then, plasticity represents the relation between the first recorded point of the mentioned equilibrium zone and the number of epochs required to reach that point.

These two properties define the learning capacity of a model under specific CL conditions. Intuitively we observe the contradiction between them, and therefore, it is important to reach a reasonable trade-off [21], where the model can acquire new knowledge without considerable performance compromises.

### 3.3. Matrix A

A, as previously mentioned, is formed by the gradient vectors collected during the training phase of the former task. In a per-layer configuration, this translates to an $A\in R^{nxm}$ matrix for each layer, formed by the gradient vectors used to modify layer parameters in each backpropagation step; thereafter, the number of steps is defined by the dataset and batch sizes. To minimize *m*, we trained with a relatively small dataset and further reduced the dimensions of A by subdividing it with a batch size of four, as Table A1 summarizes, resulting in $A\in R^{nx125}$.

### 3.4. Fisher Information Matrix

In supervised learning, training AI models means finding a set of parameters (θ) for which, given the input set (x), the model is capable of closely approximating the output (y). In other words, given the training set S(x,y), the goal is to minimize the objective function,

(6) $$h\left(\theta \right)=\frac{1}{∥S∥}\sum_{(x,y)\in S}L\left(y,f\left(x,\theta \right)\right),$$

where L(y,z) is a loss function that measures the difference between the model’s prediction *z* and target *y*. This applies for any loss function, including cross-entropy loss $L\left(y,z\right)=-\sum_{j}y_{j}\log\left(z_{j}\right)$, which is important for generating the Fisher matrix. Differentiating the loss with respect to θ, we obtain the respective gradients $\nabla y\log\left(f(x,\theta )\right)$ [22].

The Fisher matrix of the model’s learned distribution with respect to θ is

(7) $$F\left(\theta \right)=\underset{P_{x,y}}{E}\left[\nabla \log p\left(x,y|\theta \right)\nabla \log p{\left(x,y|\theta \right)}^{T}\right],$$

and since p(x,y|θ)=p(y|x,θ)p(x), where p(x) does not depend on θ, then ∇logp(x,y|θ)=∇logp(y|x,θ)+∇logp(x)=∇logp(y|x,θ), where the input *x* comes from an independent target distribution, with p(x) as its density function. Then the Fisher matrix can be expressed:

(8) $$F\left(\theta \right)=\underset{x∼q(x),y∼p(y|x,\theta )}{E}\left[\nabla_{\theta }\log p\left(y|x,\theta \right){\left(\nabla_{\theta }\log p\left(y|x,\theta \right)\right)}^{T}\right],$$

which represents the Fisher information matrix formed by the loss gradients produced while learning the model’s parameter distribution $P_{x,y}\left(\theta \right)$ using the cross-entropy loss [23]. This means that the Fisher matrix is the Hessian matrix of the cross-entropy loss [24].

Regularization-based CL approaches like EWC use the diagonal of the Fisher matrix to penalize the learning of new tasks [14]. In this article we will use the same diagonal to build the projector matrix for OWM.

### 3.5. Low-Rank Approximation (LoRA)

Due to computational complexity, conventional machine learning methods can become inapplicable when dealing with large matrices. Traditionally, data reduction techniques are used to mitigate this issue, which should retain most of the intrinsic information of the original data. Low-rank approximation achieves this by decomposing the original nxm matrix A into two lower-rank (rank-k) matrices [25],

(9) $$A_{\mathrm{mxn}}\approx B_{\mathrm{mxk}}C_{\mathrm{kxn}}$$

The optimal *k* for LoRA is typically obtained by minimizing the Frobenius norm between A and its approximation $A_{k}$. Then $A_{k}=argmin_{rank(A_{k})=k}{∥A-A_{k}∥}_{F}$, where the Frobenius norm, ${∥M∥}_{F}$, of a matrix $M=(M_{\mathrm{ij}})$ is given by ${∥M∥}_{F}=\sqrt{\sum_{i,j}M_{\mathrm{ij}}^{2}}$, and then $A_{k}$ can be formulated as a Singular Value Decomposition (SVD) of A. Since the SVD of $A\in R^{nxm}$ is $A={\mathrm{UDV}}^{T}$, where U and V are orthogonal, $D=diag(\sigma_{1},\ldots ,\sigma_{r},0,\ldots ,0)$, $\sigma_{1}\geq \ldots \geq \sigma_{r}>0$, and r=rank(A) [26].

Rank *k* can be approximated following two approaches: fixed-rank approximation inputs a rank and searches for a matrix that minimizes the Frobenius norm, while fixed-precision approximation uses a predefined error tolerance ϵ in the search for rank *k* such that $A_{k}=argmin_{rank(A_{k})=k}{∥A-A_{k}∥}_{F}\leq \epsilon$ [25]. In the present work we implement the latter approach, considering a permissible error value of 20%, which, on average, among all layers, returns k=8, reducing from $A\in R^{n\times 125}$ to $A_{k}^{a}\in R^{n\times 8}$ and $A_{k}^{b}\in R^{8\times n}$.

### 3.6. Null Space Eigenface (NEig-OWM)

Many approaches have been developed to balance the stability–plasticity trade-off. Architecture-based-approach researchers concluded that deeper models show better plasticity and wider models better stability [27]. Normalization-based approaches introduce normalization layers with different strengths to maintain both high stability and plasticity [18]. Optimization-based techniques focus on enhancing the parameter updating rules by either introducing regularization terms or modifying the gradients applied to the model. OWM belongs to the second category, since it influences the parameter updating $\theta =\theta +\eta P\nabla_{\theta_{CL}}$. NEig-OWM, however, updates the parameters while directly targeting the stability–plasticity trade-off [28], including in its projector $P_{Nl}$, a specific term for the respective property,

(10) $$P_{\mathrm{Nl}}=P_{l}\left(n\right)+\omega N_{l}N_{l}^{T}$$

where $N_{l}$ refers to the null space formed by the eigenvectors whose corresponding eigenvalues are equal to zero $N_{l}\left(\lambda =0\right)=\left\{A_{l}\left(i\right)|\lambda_{lr}\left(i\right)=0\right\}$, $P_{l}$ refers to the orthogonal projector matrix formed by the eigenvectors whose eigenvalues are different from zero, and ω is a hyperparameter that regulates new learning. $P_{l}$ increases the model’s stability by projecting new knowledge orthogonally to previous knowledge, and the second term enhances plasticity by favoring learning in previously unused areas of the model.

In our implementation we use the linear algebra PyTorch (version 2.7.1, from PyTorch, San Francisco, CA, USA) function torch.linalg.eig [29] in the decomposition of $A=Vdiag\left(\Lambda \right)V^{-1}$, where the eigenvectors $V\in R^{nxn}$ and the eigenvalues $\Lambda \in R^{n}$. The null space is extracted by filtering eigenvectors whose corresponding eigenvalue is below 1 × $10^{-30}$, while the remaining vectors form the non-null space. The projector is built with these spaces and a small constant value (1 × $10^{-3}$) for the regularization parameter ω according to Equation (10).

### 3.7. Experimental Setup

**Figure 2:**
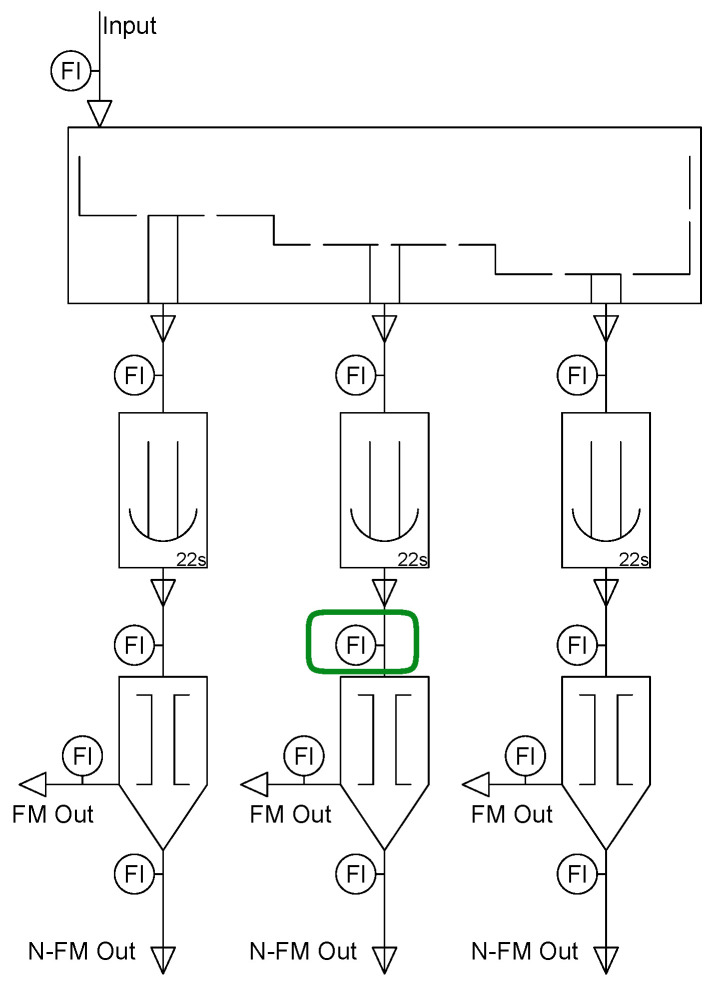
Flow diagram of sorting process. The input enters the sieving machine, followed by three conveyor belts, which are each followed by a magnetic sorter, with corresponding Flow sensors (Fl) in several sections of the process. Note: Adapted from “The Artificial Neural Twin—Process optimization and continual learning in distributed process chains” by Emmert et al., 2024 [3]. Licensed under CC BY 4.0.

As mentioned previously, for our experiments, we simulated a section of a bulk material recycling process using the physics engine of the Unity game development framework (version 2021.3.19f1, from Unity Technologies, San Francisco, CA, USA) [30]. This controlled environment provides us with a material stream that emulates the complexity of a real material sorting task. Inside the simulated process, we begin with a machine (“siever”) that sorts materials by size (Small, Medium, Large). In each outlet, we placed conveyor belts that emulate the delay between sorting machines, and after each of them, we placed magnetic sorters, which sort materials into metallic and non-metallic per the flow diagram in Figure 2.

Within our simulation, each device is independent and insulated from the information network. Then the ANT allows this to be converted into a machine learning distributed system, providing a platform for all devices to exchange information for the process to be optimized and for the models to be trained through CL.

**Figure 3:**
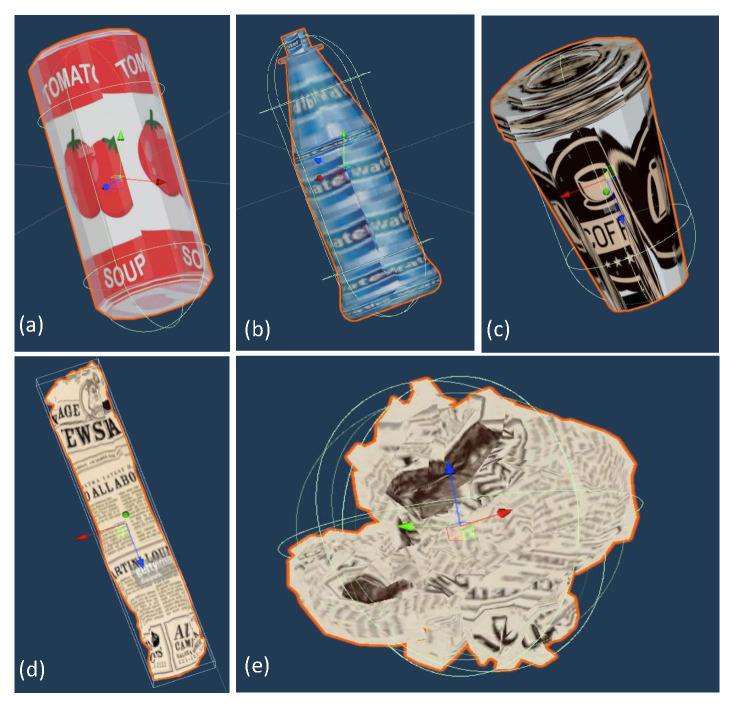
Objects present in the sorting process: (**a**) Can, (**b**) Bottle, (**c**) Coffee cup, (**d**) Paper roll, (**e**) Paper ball.

The AI sensor in this study is set above the middle conveyor belt, as highlighted in the diagram, which is a location reached by the full set of objects involved in the process. Figure 3 depicts the mentioned set, from which we initially removed the Cans (a) that will later be used as a continual learning task. In Appendix C we provide an example of the sensor’s area of detection, with the corresponding identification of the mentioned materials.

Table 1 summarizes the material properties set in our simulation environment. These properties are used by the physics engine to represent phenomena like magnetism and gravity, which are important for the functioning of the simulated sorting machines as well as for the material traffic through the process, including some of its common problems (i.e., impurities in sorting outputs, materials being dropped from conveyor belts, or getting stuck in machine parts), which amount to a realistic process complexity level. For completeness, the mentioned table includes two additional material types (FeM and NFeM caps), which are part of the overall process, sorted as small classes by the siever. Due to their dimensions (diameter less than 2.5 cm), they cannot be detected on a moving conveyor belt by the vision sensor subject of this study; therefore, they were excluded from the simulation data presented in Table 2.

Within the simulation, we configured the sensor’s area of vision such that 1080 × 512 pixel images correspond to one-second mass flow in the process. For this we consider the speed (2 m/s) and width (1.2 m) of the conveyor belts to determine the area of the belt that is displaced each second (area of 2 × 1.2 m^2^); the counts of objects contained in this area represent the one-second mass-flow window, which we later enclose in the mentioned image size. This allows for calculation of mass flow at resolutions of per second or more. Table 2 shows the material distribution throughout the different datasets collected using this data configuration.

### 3.8. VGG11 Model

As previously introduced, our vision sensor is powered by a VGG11 (architecture as in VGG11-A [31], with conv2 layers given the 2D data) trained as a Single-Shot MultiBox Detector with input resized to 300 × 300 pixel images (SSD300), which is the basis for extracting low-feature maps. To classify different objects with similar low features, as in our use case (i.e., distinguishing Cans from Coffee cups), a high-feature map extractor is used on top [32], followed by a set of convolutional layers that map the location and class of the object to the prediction. The architectures of the additional layers are described in Table 3.

To avoid deviating from the goal of this research, further details of the model training and its parameterization are included in Table A1 in Appendix B.

### 3.9. Test Cases

Even though continual learning is usually performed online (during process operation), the initial training (Training dataset in Table 2) of the AI model is typically conducted offline. During this period we collect the training loss gradients, such that at the end of each model layer, we obtain a matrix A where the rows are given by the number of layer parameters, and the columns by the number of training samples. These matrices represent the original task’s knowledge space, which should be preserved during CL [6].

We extract all representations from the respective A of each layer. Since we used cross-entropy loss for the initial training, the Fisher matrix, per Equation (8), will correspond to A multiplied by its transpose, from which we will extract and store only the diagonal to build the OWM projector. For LoRA approximation of A, we apply SVD, and then extract and store the lower-rank ${\mathrm{LoRA}}_{B}=U_{k}$ and ${\mathrm{LoRA}}_{C}=D_{k}*V_{k}^{T}$, as explained in Section 3.5. In NEig-OWM we divide matrix A into two matrices per Equation (10), where we extract the eigenvectors and eigenvalues, separating the null and non-null space, and store their corresponding matrices.

The objective of our investigation is to evaluate alternative representations of matrix A that better fit hardware and networking constraints. Therefore, the model, dataset, loss function, optimizer, and use of OWM as the CL method remained constant throughout our experiments. Meanwhile, we tuned the parameters (Validation dataset in Table 2) of the representation approaches to better approximate the original A.

Algorithm 1 summarizes the CL training process (CL dataset in Table 2) used in our research. Here, each image is used for training only once, and thereafter, it can be used to measure convergence speed. This algorithm also shows our implementation of OWM in a per-layer configuration, which reduces the memory required to project a gradient vector.
**Algorithm 1:** CL Training Loop
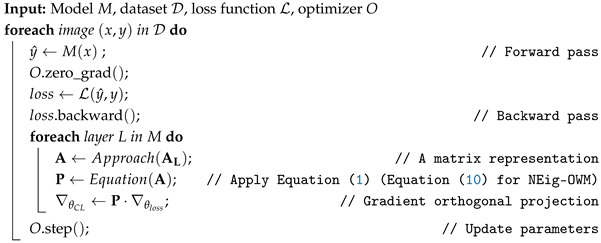


Here we apply the approach in turn to extract the specific matrix representation of A, and instead of the larger matrix *P* materializing in memory, we multiply the gradient by each term of Equation (1), reducing the load in memory from $P^{\left[n\times n\right]}\cdot \nabla_{\theta_{CL}}^{\left[n\times 1\right]}$ to $p_{1}^{\left[n\times m\right]}\cdot p_{2}^{\left[m\times n\right]}\cdot \nabla_{\theta_{CL}}^{\left[n\times 1\right]}$, where $p_{1}$ and $p_{2}$ represent the terms in the projector’s equation, and n≫m. This exempts us from storing matrix P in memory, instead storing the smaller matrix A. Afterwards, as is typical, the modified gradients are used to update the model’s parameters.

Per Algorithm 1, OWM was used to learn the new task (detecting and classifying Can objects), and we recorded test performance metrics (CL-Testing dataset in Table 2) to determine the efficiency of the different approaches during CL. We included the full matrix A and the pure Stochastic Gradient Descent (SGD) as top and bottom lines of the benchmark used to assess the performance of the tested approaches.

The Results section will summarize the scores obtained in our experiments on the described as well as standard metrics, such as F1 score, precision, recall, and individual metrics drawn from a confusion matrix.

## 4. Results

**Figure 4:**
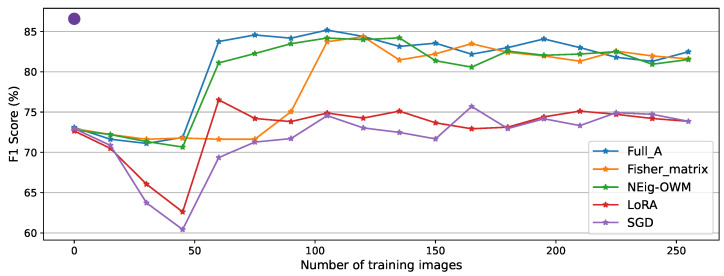
The model’s F1 score after training for the original task on test data (initial objects only), denoted by the big dot. The model’s F1 scores during CL with all approaches on CL test data (all objects) recorded every fifteen CL training images.

As previously explained, the AI sensor is originally trained to detect four objects (Bottle, Coffee cup, Paper ball, Paper roll), which we refer to as the original task. Figure 4 shows its performance (F1 score of 86.70%) after the mentioned training, shown by the big dot (Testing dataset in Table 2) set at position zero in CL training images.

For every approach, we began CL with an evaluation (F1 score of 72.82%) on CL test data. The decrement in performance compared to the original task is caused by misclassifications in the new task. Then, we trained the model on gradients generated one image at a time, with evaluations on the test dataset every fifteen training images. We stopped recording after 250 images instead of using the full CL-Testing dataset, given that at this point all the tested approaches had clearly converged. This highlights the convergence speed that an optimization-based CL algorithm such as OWM can reach, within the scope of our experimental setup.

Among the approaches, as expected, the best performance was recorded while using the full A matrix, with which the model managed to learn the new task after only 60 training images, reaching 85.17% as its maximum performance, followed by a minimal forgetting phase to end with a final score of 82.64%. Similar behavior was recorded for the NEig-OWM approach, with maximum and final F1 scores of 84.24% and 81.45%, respectively. The Fisher matrix comes in third, learning the new task after 105 images, with its maximum at 84.41% and a final performance of 81.45%, exactly the same as NEig-OWM. LoRA and SGD show catastrophic forgetting after the beginning of CL, as shown by the drops in their curves; this is even more clearly visible in their corresponding precision curves depicted in Figure A5 and Figure A7, respectively, where the per-object performance is shown. Afterwards, they learned the new task with increased overall performance, which is still comparatively low, with both finishing the experiment with an F1 score of 73.84%.

**Figure 5:**
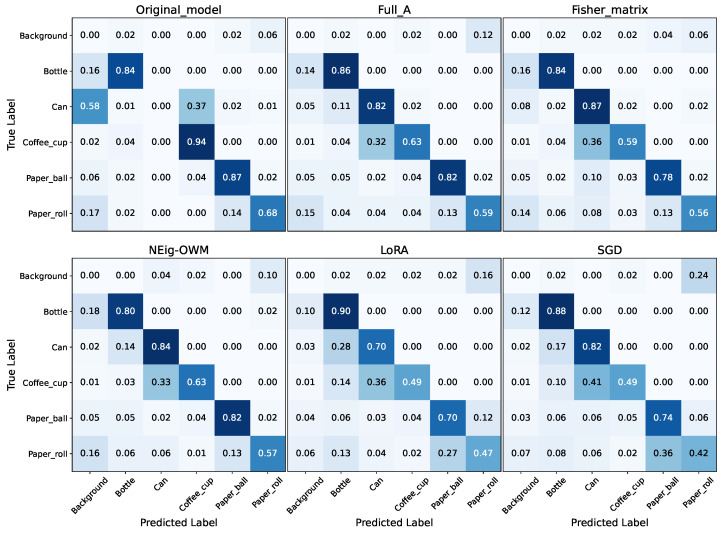
Confusion matrix of the original model in the original task, followed by confusion matrices after CL, with the model trained using every approach and evaluated on CL test data.

The described results are confirmed by the confusion matrices in Figure 5, where, once again, the full A matrix’s performance is the highest, while LoRA’s and SGD’s are the lowest. In the upper-left corner, we have the confusion matrix of the original model (before CL) on the new task, where the decrement in performance due to false-positive classifications is clearly visible. This is no longer the case after finishing CL (all other confusion matrices), where, despite the remaining cases of misclassification between the Can and Coffee cup classes, it is observed that the model can perform the new task properly. The unavoidable forgetting due to CL is present in all classes as a minimal increment in false-positive scores, and as before, LoRA and SGD have the highest rates, showing percentages higher than 15% for false-positive classifications of almost every class.

**Figure 6:**
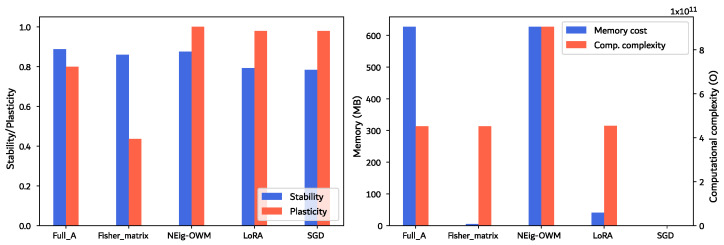
Approach comparison in terms of stability–plasticity trade-off (**left**), calculated according to the respective equations defined in Section 3.2. Memory cost in disk space for the algorithm data, and computational complexity calculated for one backpropagation step (**right**).

Figure 6, on the left, depicts the stability–plasticity scores of the tested approaches. Given the single-CL-task scenario, the forgetting measure is simply calculated as the difference in F1 scores before and after CL (both in Testing dataset in Table 2) according to Equation (2); afterwards, we use Equation (4) to calculate stability measures. For the comparison of memory and complexity requirements (Figure 6, right), memory is defined by the cost in disk storage of the full set of matrices (stored using the Shelve module of Python, version 3.10.13, from Python Software Foundation, Fredericksburg, Virginia, USA, which stores data as persistent dictionary-like pickled objects known as Shelfs in “.db” file format) required to perform CL on the entire model for each approach. Meanwhile, complexity values correspond to the number of matrix-multiplication operations required for the orthogonal projection of the gradients corresponding to a backpropagation step. We collected this data aiming to provide a clear view of the additional requirements that each approach would introduce into a system already in operation.

Here, the minimum is obtained with SGD, which represents the approach with no implementation of CL. Therefore, no memory or additional computations are required. SGD is followed by the Fisher matrix, where the storage of only the matrix diagonal gives a marked advantage in memory requirements compared to the others. From the diagonal, we approximated $A\in R^{nxm}$, which translates to complexity equal to that when using the full A matrix, which is also the case for LoRA. Since NEig-OWM divides the A matrix into two and generates a projector for each section, this translates into the same storage requirements as full A, but double the computational operations.

For the stability–plasticity trade-off in Figure 6 (left), the average stability through classes shows similar behavior for full A, the Fisher matrix, and NEig-OWM, while a lower score is recorded for LoRA and SGD due to greater forgetting. Meanwhile, plasticity is calculated exclusively for the new task. High plasticity is shown by LoRA, SGD, and NEig-OWM, where the former two are highly plastic due to lower stability, while NEig-OWM remains high in both properties thanks to its mentioned characteristics that specifically address this trade-off. The higher stability using the full A matrix is contrasted by lower plasticity; this effect is even greater when using the Fisher matrix, where the plasticity drops to 40%.

Its low memory requirements and complexity, plus its high stability, despite its lower plasticity, make the Fisher matrix the minimum and best-performing approach within the scope of our experiments. Therefore, in this last section, we will focus mostly on its results.

**Figure 7:**
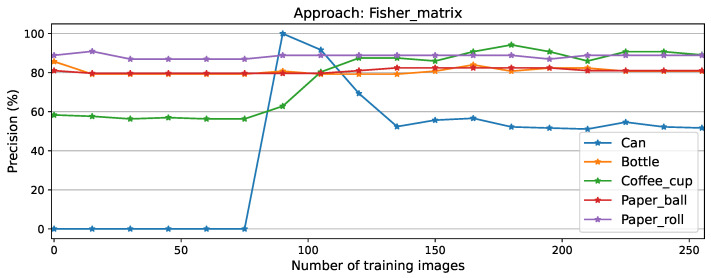
Per-class precision scores for Fisher matrix approach.

**Figure A1:**
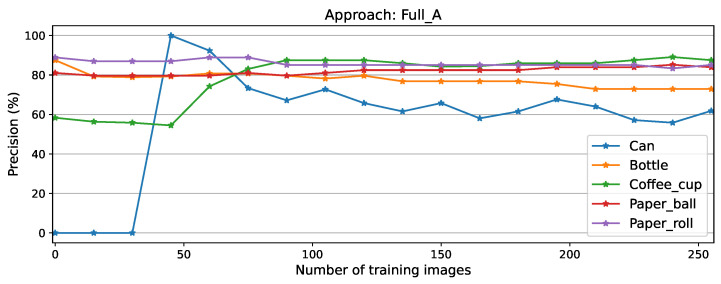
Per-class precision scores for full A matrix approach.

Per-class precision for the Fisher matrix approach is shown in Figure 7. Here, the process of learning the new task is clearly visible, with an initial peak in performance at 90 training images due to the model’s double detection (Cans labeled as Coffee cups and Cans simultaneously), which was later overcome, converging at 56.72% precision on the new task, which was not the highest score but was the most steady convergence compared to equivalent plots for other approaches, displayed in Figure A1, Figure A3, Figure A5, and Figure A7.

**Figure 8:**
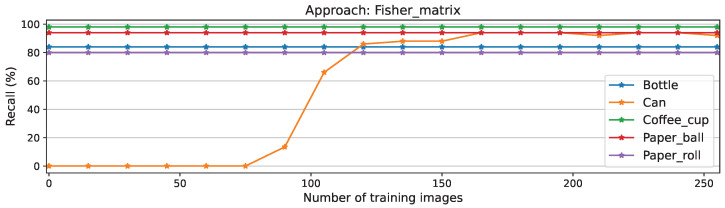
Per-class recall scores for Fisher matrix approach.

**Figure A2:**
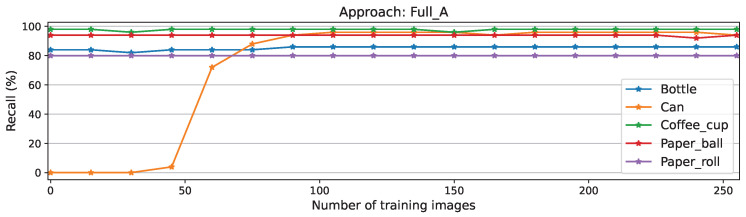
Per-class recall scores for full A matrix approach.

**Figure A3:**
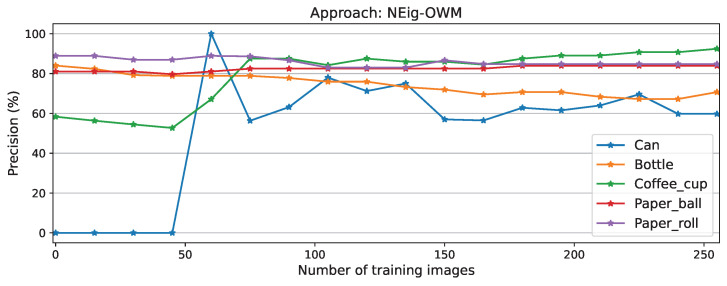
Per-class precision scores for NEig-OWM approach.

**Figure A4:**
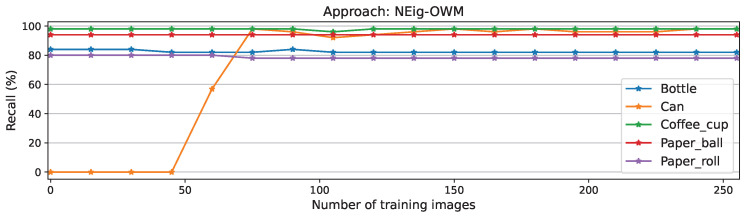
Per-class recall scores for NEig-OWM approach.

**Figure A5:**
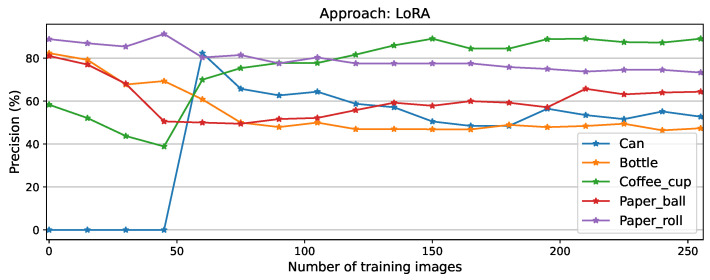
Per-class precision scores for LoRA approach.

**Figure A6:**
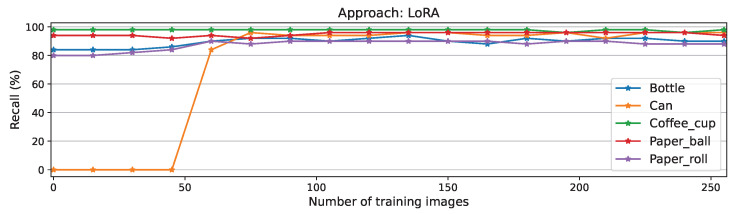
Per-class recall scores for LoRA approach.

**Figure A7:**
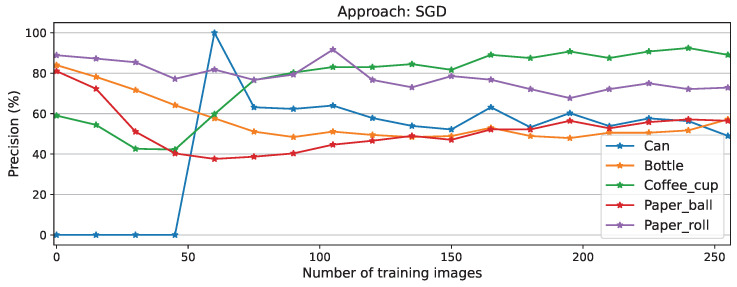
Per-class precision scores for SGD approach.

**Figure A8:**
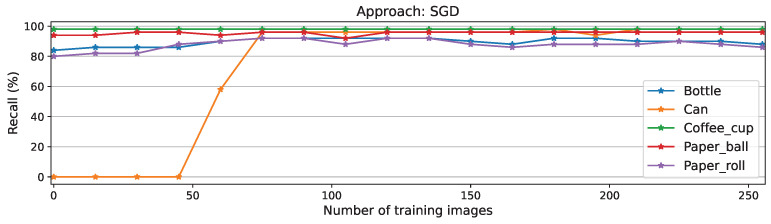
Per-class recall scores for SGD approach.

The stability of the approach is demonstrated here by the nearly constant scores of known classes, except for the class Coffee cup, which initially included falsely classified Cans. This issue is clarified in the recall plots in Figure 8, where false positives have no influence and therefore all-other-class scores remain constant, while the new-class score improves. This plot also shows the longer time before convergence for this approach compared to full A, NEig-OWM, LoRA, and SGD, depicted in Figure A2, Figure A4, Figure A6, and Figure A8, respectively.

As an initial example of how the calculated complexity maps to the algorithm’s latency, we recorded the loop time (including forward pass, backpropagation, and CL, in combination with the Fisher matrix approach to update the model) of CL on the described version of VGG11 (disk space 151.14 MB) running in a lower-capacity machine (HP EliteBook 840 G8, processor Intel Core i7, 3.00 GHz, no GPU available). The average loop time recorded over the 250 images (1080x512pixels and 20.48 KB each) shown in our previous experiments was 2.58 s.

## 5. Discussion and Conclusions

The LoRA approach did not perform much better than plain SGD, which we understand is due to the highly minimized rank when approximating A, which is predicated on $A_{k}$ losing parameters that are important for defining the original task’s knowledge space. Given that our objective is to find the least expensive solution, we considered that there was no need to further search for a higher-rank approximation. Moreover, the diagonal of the Fisher matrix proved to be a less expensive yet effective option. Therefore, we conclude that it is a viable solution for large models installed on devices with relatively tight hardware constraints, as depicted in Figure 4 and confirmed by its plasticity score in Figure 6.

We presented full A as a benchmark; however, this is still an option for scenarios with medium to large models, where the hardware is not considerably restrictive and a faster learning speed is required. NEig-OWM is the approach with the highest hardware demands, which makes it suitable for situations with no restrictions or when hardware constraints are overcome by implementing small models, which, however, present bigger parameter overlaps between tasks. Here, the approach’s direct regard for the stability–plasticity trade-off allows for greater segregation of the non-overlapping areas of the model, which translates into greater control over forgetting while learning new tasks.

Despite the poor performance of the LoRA approach, we consider it a viable candidate for settings where the cross-entropy loss (required for the Fisher matrix) is not applicable, keeping in mind the extensive optimization of the rank selected. This amounts to the conclusion that the distinct characteristics of the approaches tested make them plausible options for scenarios where one of them has been proven ineffective.

Our future research will involve CL on a vision sensor, powered by a large object detection model with a Fisher matrix representation of matrix A, and the implementation of NEig-OWM on a small machine model (i.e., the input/output prediction model of a magnetic sorter). We will insert both models into the ANT network so that we can analyze the performance of CL using QC gradients and DF residuals as training stimuli.

## Figures and Tables

**Table 1:** Material properties set in simulation environment. Size class is used by siever to sort materials by size, and ferromagnetic class is used by magnetic sorter to separate Ferromagnetic (FeM) from Non-Ferromagnetic (NFeM) materials.

Name	Mass [kg]	Ferromagnetic Class	Size Class
Paper roll	0.0090	NFeM	Large
Bottle	0.0180	NFeM	Large
Coffee cup	0.0093	NFeM	Medium
Paper ball	0.0090	NFeM	Medium
Can	0.0149	FeM	Medium
FeM cap	0.0007	FeM	Small
NFeM cap	0.0005	NFeM	Small

**Table 2:** Material content of different datasets used in experiments.

Dataset	Materials	Number of Images
Training	All, excluding Cans	500
Validation	All, excluding Cans	250
Testing	All, excluding Cans	300
CL	Cans only	300
CL-Testing	All	300

**Table 3:** Additional layers included in the VGG11 model structure.

High-Feature Map		Prediction Convolutions		
conv2	256	conv2	512	Location
conv2	512	conv2	1024	
conv2	128	conv2	512	
conv2	256	conv2	256	
conv2	128	conv2	256	
conv2	256	conv2	256	
conv2	128	conv2	512	Class
conv2	256	conv2	1024	
		conv2	512	
		conv2	256	
		conv2	256	
		conv2	256	

**Figure A9:**
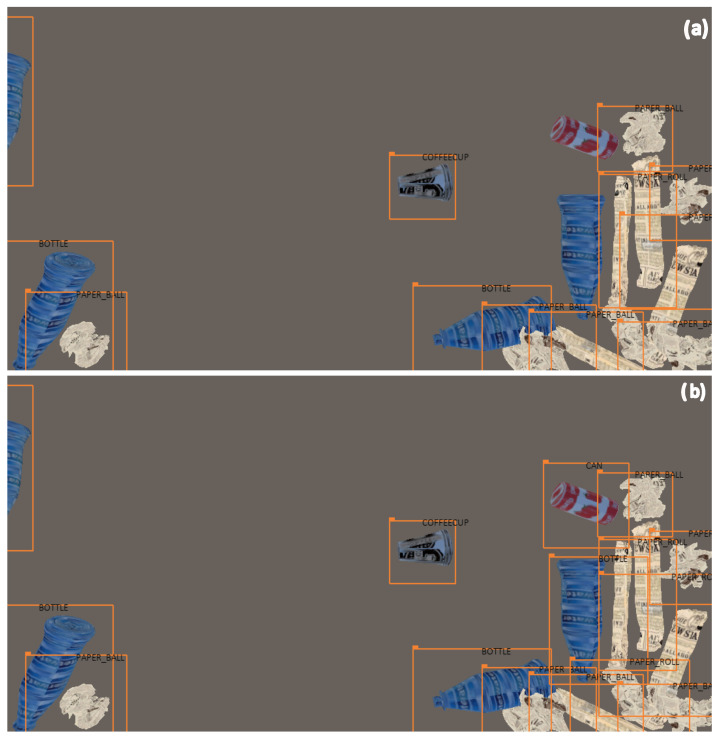
Sensor recording one-second mass flow of the material stream transported by a conveyor belt. (**a**) Before CL, (**b**) after CL.

## Data Availability

The data presented in this study are available on request from the corresponding author due to institutional policies.

## Abbreviations

The following abbreviations are used in this manuscript:

ANT	Artificial Neural Twin
AI	Artificial Intelligence
AR1	Architectural and Regularization
CL	Continual Learning
DF	Data Fusion
EWC	Elastic Weight Consolidation
Fl	Flow sensor
FM	Forgetting Measure
FeM	Ferromagnetic
NFeM	Non-Ferromagnetic
IIoT	Industrial Internet of Things
LoRA	Low-Rank Approximation
lr	Learning Rate
MEC	Mobile Edge Computing
NN	Neural Network
NEig-OWM	Null Eigenface-Orthogonal Weight Modification
OWM	Orthogonal Weight Modification
QC	Quality Control
SGD	Stochastic Gradient Descent
SSD	Single-Shot MultiBox Detector
SVD	Singular Value Decomposition
TDM	Task-Aware Dynamic Masking
VGG11	Visual Geometry Group 11

## Appendix B. Hyperparameters Used for Training and CL

The standard loop is use for model training with additional steps for continual learning, as explained in Algorithm 1; the Validation dataset and greedy-search algorithm were used to determine the hyperparameters of the distinct training phases, which are displayed in Table A1, highlighting that, for CL, images are provided one at a time (Batch size 1) until the model reaches convergence (training stopped at 250 images).

**Table A1:** Hyperparameters set for initial training and CL phases.

	Parameter	
Name	Training	CL
Batch size	4	1
Epochs	100,000	250
Learning rate (lr)	1 × $10^{-3}$	3 × $10^{-3}$
lr decay at	[1700, 5000, 10,000]	Non
lr decay to	0.1	Non
Weight decay	5 × $10^{-4}$	5 × $10^{-4}$
Momentum	0.9	0.9
Optimizer	SGD	SGD
Loss futn.	Cross-entropy	Cross-entropy
Alpha	Non	1 × $10^{-4}$
Pre-training	No	Yes

## Appendix C. Sensor Reading

Figure A9 depicts the conveyor belt area that encloses one-second material mass flow recorded by the AI sensor in this study. Here we recorded the sensor’s detection before CL (a), when the sensor did not know about the new task (Can), and after it learns this task (b).
